# The effect of khat chewing on sexual desire among adults in North East Ethiopia: a propensity score-match analysis

**DOI:** 10.3389/fpubh.2025.1470982

**Published:** 2025-07-03

**Authors:** Temesgen Gebeyehu Wondmeneh, Fikre Enquselassie

**Affiliations:** ^1^Department of Public Health, College of Medical and Health Science, Samara University, Semera, Ethiopia; ^2^Department of Preventive Medicine, School of Public Health, College of Health Science, Addis Ababa University, Addis Ababa, Ethiopia

**Keywords:** effect, khat, chewing, sexual desire, adults

## Abstract

**Background:**

Although the number of khat chewers has increased from time to time in Ethiopia, the direction (increased or decreased) of khat chewing on sexual desire is not well known due to the absence of adequate studies. Therefore, this study aimed to assess the effect of khat chewing on sexual desire in adults.

**Methods:**

A comparative cross-sectional study was conducted in Logia town. A total of 334 study subjects, comprising 167 khat chewers and 167 non-khat chewers, were included in this study. House-to-house data were collected using the purposive sampling method. To estimate the effect of khat chewing on sexual desire, propensity score matching analysis with a logit model was used to find the average treatment effect (ATE) on the treated and untreated groups. The matching quality was checked statistically and graphically. Logistic regression was used to determine factors associated with sexual desire.

**Results:**

About 54.5% of respondents had poor sexual desire. Among khat chewers, 28.1% had poor sexual desire. According to logistic regression analysis, sexual desire was 2.8 times higher in adults aged 18–25 years (AOR = 2.8, 95% CI: 1.02–7.8). Sexual desire was lower in traders (AOR = 0.32, 95% CI: 0.15–0.69), low-income level (AOR = 0.37, 95% CI: 0.16–0.86), and cigarette smokers (AOR = 0.4, 95% CI: 0.2–0.73). There was no significant association between khat chewing and sexual desire (AOR = 0.91, 95% CI: 0.53–1.5). In propensity score matching analysis, the average treatment effect (ATE) on adults who chewed khat was 18.9%, meaning an average increase in sexual desire of 18.9%. The treatment impact on the treated group (ATT) was found to be 24.2%, indicating that 24.2% increased sexual desire among the treated groups (khat chewing).

**Conclusion:**

More than half of adults had poor sexual desire. Logistic regression analysis revealed that sexual desire is moderated by age, occupational status, income level, and use of cigarettes. In the propensity score matching analysis, khat chewing can significantly increase the levels of sexual desire.

## Introduction

Khat is a stimulant extracted from the fresh leaves of the evergreen shrub *Catha edulis*, which is indigenous to parts of East Africa and the Arabian Peninsula (1). In the isolated khat (*Catha edulis*) extract, cathine and norephedrine are the primary alkaloids, with respective compositions of 81.3 and 17.2% (2). Khat is a substance that is widely used recreationally in the Horn of Africa and the Middle East (3). In Ethiopia, khat chewing is a common practice, with 15.8% of people chewing (21.1% of males and 9.4% of females). Khat chewing practice was more common in the eastern part of Ethiopia, such as the Afar, Dire Dawa, Harari, and Somalia regions (4, 5). Using EDHS 2016 data, the prevalence of khat chewing ranged from 23.6 to 61.9% (5, 6).

Studies have reported that substance use, such as alcohol, khat, and cigarette, increased sexual behaviors (7–9). Khat chewing increased risky sexual behavior (10–12). A study in Kenya and Uganda showed that khat can alter one’s libido and sex motivation. Khat chewing encourages women to engage in sex work, leads to sexual violence, and spreads STDs like HIV. Men who chew khat are more likely to commit rape (13). In a study conducted in Yemen, 81% of erectile dysfunction cases were khat chewers. The most common erectile dysfunction was psychological, especially in young people. However, no significant association was noted between Khat chewing and psychological erectile dysfunction (14). In an *in vitro* study of female rats, the release of alkaloids from dried khat raised sexual motivation and estradiol levels in female rats (2). In a study of male rates, higher doses of khat inhibited sexual behavior, whereas a low dose of khat with concurrent administration of ethanol enhanced sexual motivation and arousal (15). Chronic khat use in humans showed decreased libido or erectile dysfunction (16). A review of studies regarding the impact of khat chewing on reproductive function showed that regular khat chewing appears to reduce human libido and may even cause sexual impotence (17–19).

The number of khat chewers in Ethiopia is increasing from time to time; however, there is no clear direction (increase or decrease) in the relationship between khat chewing and sexual desire from previous reports. Thus, this study can contribute to solving this controversy reported in the literature. This study provides clinicians with insights in to considering and establishing mechanisms for solving problems in the future and to develop possible recommendations. The findings of this study will also inform harm reduction programs, and policymakers may use the data to establish regulations and laws regarding the issue.

## Methods

### Study design and site

A community-based comparative cross-sectional study was conducted among khat chewers and non-chewers to assess the effect of khat chewing on sexual desire among adults in Logia town, Afar region, northeast Ethiopia. The study was conducted from May 10 to June 1, 2016. The region is dry with high temperature ranging from 35°C to 45°C. According to the Afar Regional Health Bureau, the total population of Logia town is 22,960; of them, 12,123 (52.8%) were adults. There is no khat cultivation in the region.

### Inclusion and exclusion criteria

Adults aged 18–49 years were included in the study regardless of their sex. Adults with any chronic comorbidity, such as diabetes mellitus, hypertension, or mental health problems, and those on medication were excluded because these comorbid diseases and their medication can affect their sexual desire.

### Sample size determination and sampling procedures

The sample size was calculated using the double population proportion formula using Epi Info version 7. Taking the proportion of increasing sexual desire among khat chewers to be 20.6% (20) and assuming a difference of 11.4% between khat chewers and non-chewers, the ratio of khat chewers to non-chewers is 1:1, with an alpha 5% significance level and a power of 80%. The estimated sample size was 302 (151 for each group). Adding a non-response rate of 10%, the required sample size for the study was 334. Then study subjects were selected from house to house using purposive sampling methods until the required sample sizes of khat chewers and non-chewers were obtained.

### Operational definition and outcome measurement

**Sexual desire**: Sexual desire (libido) is the feeling of wanting to have sex or engage in sexual activities. Thirteen items of dyadic sexual desire (items 1–9) and solitary sexual desire (items 10–13) were used to measure sexual desire. Dyadic desire describes an interest in or a wish to engage in sexual activity with another person, while solitary desire describes an interest in engaging in sexual behavior by oneself. The validity of the items was first provided to sexology researchers and clinicians, who rated the validity and clarity of the items. Internal consistency was estimated by Cronbach’s alpha, which revealed coefficients of 0.86 for dyadic sexual desire and 0.96 for solitary sexual desire (21). There were 7 possible responses in the Likert scales of the first two items: not at all (0 score), once a month (1 score), once every 2 weeks (2 score), once a week (3 score), twice a week (4 score), 3 to 4 times a week (5 score), once a day (6 score), and more than once a day (7 score). The Likert scale of the next 11 items had 8 possible responses: (no desire) 0 1 2 3 4 5 6 7 8 (strong desire) (21). The sexual desire inventory tool is provided in Supplementary file 1. The participant’s Likert scale score for the 13 sexual desire items was summed up to obtain the sexual desire score. Participants were considered to have poor or decreased levels of sexual desire if their total score was at or below the median; otherwise, they were considered to have good or increased levels of sexual desire.

**Khat chewing**: an adult who chews khat currently or within the last 30 days.

### Variables

The outcome variable was sexual desire (poor and good sexual desire). Socio-demographic variables were age, sex, marital status, occupation, education, ethnicity, religion, and income. The exposure variable was khat chewing (yes/no, frequency, and amount of khat chewing). The other substance use variables were cigarette smoking, shisha, and alcohol drinking.

### Data collection tools and methods

The data were collected using structured interview questionnaires, which consist of four parts: personal information, khat chewing (frequency and amount of khat chewing), substance use, and sexual desire. The first two were developed from an existing literature review, and the last was based on sexual desire inventory questions. First, the questionnaires were developed in English by the investigator, then translated to a local language (Afar) by experts, and finally translated back to English. A total of eight data collectors were recruited from Soloda Health Science College; four were for khat chewers, and the remaining four were for non-khat chewers. Two health officer supervisors, one for khat chewers and the other for non-khat chewers, were recruited from Samara University instructors. Because of the presence of study subjects who may not be able to speak the local language or Amharic in the study area, all selected data collectors were able to speak and read both Amharic and local languages (Afargna) perfectly, and they could translate either language. Training was given to eight selected data collectors. The objective of the study, how to fill out a questionnaire, and the ethical aspect of confidentiality were the focus of training. The questionnaires were pre-tested before data collection in Samara town, and corrections were made based on the pre-test. Due to the sensitive nature of sexual desire, the participants were interviewed in a private, secure location that had not been visited by anyone else. The principal investigator monitored the data collection process and explained if any problems arose during data collection.

### Data analysis

The data were entered into Epidata version 3.1 and then exported to Stata software version 15 for further analysis. Descriptive statistics such as percentage, frequency, median, and interquartile range were computed. The chi-square was used to assess the association between khat chewing and substance use, as well as the association between sexual desire and the frequency and amount of khat chewing. Logistic regression analyses were used to determine the association between independent and dependent variables. Variables less than 0.25 in the binary logistic regression were included in the multivariable logistic regression. Multicollinearity was diagnosed using variance inflation factor (VIF). Statistical significance was declared at *p* < 0.05 in the multivariable logistic regression.

### Propensity score and average treatment effect

In observational studies, unlike randomized clinical trials (RCTs), randomization is not feasible. As a result, there is an intrinsic imbalance in the variables that are seen, which introduces bias and affects the exposure’s causal effect.

In this study, sexual desire may not be directly affected by khat chewing; instead, it may be influenced by a variety of known and unknown factors. Therefore, using a regression statistical test is hard to examine the relationship between khat chewing and sexual desire. Regression analysis only accounts for observed variables; residual confounding bias still persists. Even after accounting for these factors in the regression model, there may still be bias in the relationship between chewing khat and sexual desire due to the distribution of factors affecting chewing khat relative to those who did not chew khat. Propensity score matching is a good approach to estimating the actual effect of khat chewing on sexual desire. Propensity score matching is a methodological technique that is intended to remove bias by matching treated (khat chewers) and untreated (non-khat chewers) adults with similar conditional probability to receive the treatment. In this study, we matched adults with non-khat chewers with adults with khat chewers with similar propensity score values. Therefore, it can be reasoned that any difference in sexual desire is solely related to khat chewing. In general, propensity score matching was employed to mitigate these possible selection biases. Variables such as age, sex, marital status, occupation, religious, ethnicity, educational status, income, smoking, alcohol, and shisha were considered for matching. The matching also considered factors significantly associated with the treatment and outcome variables. The treatment group (khat chewers) and control group (non-khat chewers) were fully matched 1:1 with weight based on a fully matching propensity score. By matching them on a variety of factors that may have an impact on the probability of khat chewing, propensity score matching aims to produce two groups that are similar. Using important factors, a logit model was constructed to predict treatment.

The treatment variable was khat chewing, represented by 1, and non-khat chewing, represented by 0.

This conditional probability, named as propensity score, was calculated using the following equation: *P* (khat chewing) = Pr (*Ti = 1/Xi*), where Ti represents if respondent i is an adult with khat chewing and Xi represents the covariates for respondent i that predict khat chewing and are thought to confound the association between khat chewing and sexual desire. The value of propensity score ranges from 0 to 1. Scores closer to 1 denote a high propensity to be affected by khat chewing, while scores closer to 0 indicate a low propensity. Then, propensity scores were assigned to each participant, and the common support assumption, which ensures that treatment observations have comparison observations “nearby” in the propensity score distribution, was checked graphically. The treated group (adults with khat chewing) was matched with the control group (adults without khat chewing) based on propensity scores. To determine the covariate balance, various matching techniques were experimented with, including nearest neighbor matching with and without replacement and radius matching with calipers set to 0.15. The “psmatch2 ate” STATA command was used to determine the average treatment effect on the population (ATE) and treated (ATT). The ATE is the average effect, at the population level, of moving an entire population from untreated to treated, while the ATT is the average effect of treatment on subjects who ultimately received the treatment (22). The assessment of match quality was based on how well the covariates were balanced between the treatment and control groups. Initially, match quality was assessed by calculating the standardized bias both before and after the matching process. This bias was determined by the percentage difference in the average values of the sample means between the treated and control groups, divided by the square root of the average sample variances in both groups. While there’s no strict threshold for standardized differences to signal an imbalance, a difference below 10% is generally considered negligible. Additionally, pseudo-R-squared and the likelihood-ratio tests were employed to evaluate the collective non-significance of all covariates in the logistic regression model of the conditional probability of treatment, both pre- and post-matching. To verify the reliability of the Propensity Score Matching results, a sensitivity analysis was performed (23). Given that the outcome variables were dichotomous, the Mantel–Haenszel statistic was used to determine if the Propensity Score Matching results were influenced by any concealed biases (24). The gamma coefficient represents the degree to which an unmeasured confounder or hidden bias might affect the allocation of the intervention between the treated and control groups. The gamma value was varied from 1 to 2 in increments of 0.05 using the mhbounds command in STATA (25).

### Ethical clearance

Ethical approval for the study was obtained from the Addis Ababa University School of Public Health and Research Ethics Committee prior to data collection. An official letter was written from the school to the respective bodies. The Afar regional health bureaus and the Logia town administration had given permission to collect data and provide information about Logia town. Written informed consent was obtained from each participant. Participation was voluntary, and they could withdraw from the study at any time. The right of each respondent to refuse or answer a few or all questions was respected. Omitting the names of the study subjects from the questionnaires helps to assure confidentiality of the information, and maximum effort was made to maintain the privacy of the respondent during the interview. The rights and autonomy of all participants were also respected.

## Results

### Sociodemographic characteristics of study participants

In this study, 334 study participants, made up of 167 khat chewers and 167 non-chewers, were recruited. Most of the study subjects (44.6%) were 18–25 years old. Males and females were nearly comparable; half were males and half were females. Unmarried participants accounted for 62%. Traders were 42.2%. Three-fourths of the study participants were Muslims. The majority of study participants were Amhara and Afar, at 49.7 and 29.6%, respectively. The percentages of participants with no education, primary education, and secondary education were 28.7, 31.7, and 29.9%, respectively. The majority of study participants (26.9%) had average income levels between 2001 and 2004 (Table 1).

**Table 1:** Sociodemographic characteristic of study participants.

Variables	Categories	N	Percentage
Age (in year)	18–25	149	44.6%
	26–30	95	28.4%
	31–35	36	10.8%
	36–40	27	8.1%
	41–49	27	8.1%
Sex	Females	165	49.4%
	Males	169	50.6%
Marital status	Unmarried	207	62%
	Married	127	38%
Occupation	Traders	141	42.2%
	Government employs	53	15.9%
	Daily labors	84	25.1%
	Students	56	16.8%
Religious	Christian	81	24.3%
	Muslim	253	75.7%
Ethnicity	Afar	99	29.6%
	Amhara	166	49.7%
	Tigray	44	13.2%
	Others	25	7.5%
Educational status	Illiterates	96	28.7%
	Primary	106	31.7%
	Secondary	100	29.9%
	Diploma and above	32	9.6%
Income (ETB)	Less than 1,000	81	24.3%
	1,000–2000	62	18.6%
	2001–4,000	90	26.9%
	4,001–6,000	49	14.7%
	> 6,000	52	15.6%

Others represented Oromo, Harari and Southern Nation Nationality people. ETB, Ethiopian birr.

### The association between baseline characteristics of study and khat chewing

Socio-demographic characteristics such as age, occupation, ethnicity, education, and income were significantly associated with khat chewing. Khat chewing was significantly associated with cigarette smoking, alcohol drinking, and shisha taking (Table 2).

**Table 2:** The association between baseline characteristics of study and khat chewing.

Variables	Categories	Khat chewing		*p*-value
		Yes	No	
		n (%)	n (%)	
Age (in year)	18–25	59 (17.7)	90 (26.9)	< 0.001
	26–30	40 (12)	55 (16.5)	
	31–35	27 (8.1)	9 (2.7)	
	36–40	20 (6)	7 (2.1)	
	41–49	21 (6.3%)	6 (1.8)	
Sex	Females	86 (25.7)	79 (23.7)	0.44
	Males	81 (24.3)	88 (26.3)	
Marital status	Unmarried	95 (28.4)	112 (33.5)	0.055
	Married	72 (21.6)	55 (16.5)	
Occupation	Traders	71 (21.3)	70 (21)	< 0.001
	Government employs	30 (9)	23 (6.9)	
	Daily labors	55 (16.5)	29 (8.7)	
	Students	11 (3.3)	45 (13.5)	
Religious	Christian	43 (12.9)	38 (11.4)	0.52
	Muslim	124 (37.1)	129 (38.6)	
Ethnicity	Afar	29 (8.7)	70 (21)	< 0.001
	Amhara	90 (26.9)	76 (22.8)	
	Tigray	30 (9)	14 (4.2)	
	Others	18 (5.4)	7 (2.1)	
Educational status	Illiterates	61 (18.3)	35 (10.5)	< 0.001
	Primary	60 (18)	46 (13.8)	
	Secondary	28 (8.4)	72 (21.6)	
	Diploma and above	18 (5.4)	14 (4.2)	
Income (ETB)	Less than 1,000	19 (5.7)	62 (18.6)	< 0.001
	1,000–2000	35 (10.5)	27 (8.1)	
	2001–4,000	51 (15.3)	39 (11.7)	
	4,001–6,000	28 (8.4)	21(6.3)	
	> 6,000	34 (10.2)	18 (5.4)	
Cigarette smoking	Yes	49 (14.7)	13 (3.9)	< 001
	No	118 (35.3)	154 (46.1)	
Alcohol drinking	Yes	42 (12.6)	24 (7.2)	0.013
	No	125 (37.4)	143 (42.8)	
Shisha taking	Yes	70 (21)	8 (2.4)	<0.001
	No	97 (29)	159 (47.6)	

Others: Oromo, Harari and Southern Nation Nationality people. ETB, Ethiopian birr.

### Sexual desire and its association with the baseline characteristics of study participants

The overall median sexual desire was 49.9 (interquartile range = 23.6). The minimum and maximum sexual desires were 3 and 94, respectively, with a range of 91. The overall mean sexual desire score was 47.9, with a standard deviation of 18.9. The median sexual desires of khat chewers and non-chewers were 48 and 51, with IQR of 32 and 18, respectively. The mean sexual desires among khat chewers and non-chewers were 46.8 and 49, with standard deviations of 20 and 17.7, respectively. The median and mean values were almost comparable, indicating that the distribution of sexual desire among participants was not skewed. About 54.5% of respondents had poor sexual desire. Poor sexual desire among khat chewers was 28.1%, while it was 26.3% among non-khat chewers. Only cigarette smoking was significantly associated with sexual desire (Table 3).

**Table 3:** The association between baseline characteristics of study participants and sexual desire.

Variables	Categories	Sexual desire		*p*-value
		Poor	Good	
		n (%)	N (%)	
Age (in year)	18–25	72 (21.6)	77 (23.1)	0.095
	26–30	51 (15.3)	44 (13.2)	
	31–35	22 (6.6)	14 (4.2)	
	36–40	17 (5.1)	10 (3)	
	41–49	20 (6)	7 (2.1)	
Sex	Females	87 (26)	78 (23.4)	0.52
	Males	95 (28.4)	74 (22.2)	
Marital status	Unmarried	116 (34.7)	91 (27.2)	0.47
	Married	66 (19.8)	61 (18.3)	
Occupation	Traders	86 (25.7)	55 (16.5)	0.053
	Government employs	28 (8.4)	25 (7.5)	
	Daily labors	46 (13.8)	38 (11.4)	
	Students	22 (6.6)	34 (10.2)	
Religious	Christian	47 (14.1)	34 (10.2)	0.46
	Muslim	135 (40.4)	118 (35.3)	
Ethnicity	Afar	49 (14.7)	50 (15)	0.33
	Amhara	96 (28.7)	70 (21)	
	Tigray	21 (6.3)	23 (6.9)	
	Others	16 (4.8)	9 (2.7)	
Educational status	Illiterates	60 (18)	36 (10.8)	0.28
	Primary	54 (16.2)	52 (15.6)	
	Secondary	50 (15)	50 (15)	
	Diploma and above	18 (5.4)	14 (4.2)	
Income (ETB)	Less than 1,000	50 (15)	31 (9.3)	0.43
	1,000–2000	31 (9.3)	31 (9.3)	
	2001–4,000	44 (13.2)	46 (13.8)	
	4,001–6,000	29 (8.7)	20 (6)	
	> 6,000	28 (8.4)	24 (7.2)	
Khat chewing	Yes	94 (28.1)	73 (21.9)	0.51
	No	88 (26.3)	79 (23.7)	
Frequency of khat chewing per day	≤ 2 times	52 (31)	46 (27.4)	0.281
	≥ 3 times	43 (25.6)	27 (16.1)	
Cigarette smoking	Yes	44 (13.2)	18 (5.4)	0.004
	No	138 (41.3)	134 (40.1)	
Alcohol drinking	Yes	35 (10.5)	31 (9.3)	0.79
	No	147 (44)	121 (36.2)	
Shisha taking	Yes	45 (13.5)	33 (9.9)	0.52
	No	137 (41)	119 (35.6)	

### Influencing factors of sexual desire

#### Logistic regression analysis

In the binary logistic regression, age (18–25 years), trade, and cigarettes smoking were significantly associated with sexual desire. When adjusting for confounding factors, age of 18–25 years, trade, income less than 100 ETB, and cigarette smoking had a significant association with sexual desire. The level of sexual desire in adults aged 18–25 years was 2.8 times higher than in those aged 41–49 years (AOR = 2.8, 95% CI: 1.02–7.8). Sexual desire among traders was 68% less than that of students (AOR = 0.32, 95% CI: 0.15–0.69). Adults with an income level of below 1,000 ETB had 63% lower sexual desire than those with an income level of greater than 6,000 ETB (AOR = 0.37, 95% CI: 0.16–0.86). Cigarette smokers had 60% lower sexual desire compared with nonsmokers (AOR = 0.4, 95% CI: 0.2–0.73). However, there was no significant association between khat chewing and sexual desire compared to non-khat chewers (AOR = 0.91, 95% CI: 0.53–1.5). Moreover, the frequency of khat chewing per day did not significantly affect sexual desire (Table 4).

**Table 4:** Influencing factors of sexual desire.

Variables	Categories	Sexual desire		COR (95%CI)	AOR (95%CI)
		Poor (reduced)	Good (increased)		
		n (%)	n (%)		
Age (in year)	18–25	72 (21.6)	77 (23.1)	3.1 (1.2–7.7)*	2.8 (1.02–7.8)*
	26-30	51 (15.3)	44 (13.2)	2.5 (0.95–6.4)	2.5 (0.9–6.9)
	31–35	22 (6.6)	14 (4.2)	1.8 (0.61–5.4)	1.77 (0.56–5.5)
	36–40	17 (5.1)	10 (3)	1.68 (0.53–5.4)	1.5 (0.5–5.2)
	41–49	20 (6)	7 (2.1)	1	1
Sex	Males	95 (28.4)	74 (22.2)	0.87 (0.57–1.34)	0.83 (0.5–1.4)
	Females	87 (26)	78 (23.4)	1	1
Marital status	Unmarried	116 (34.7)	91 (27.2)	0.85 (0.55–1.3)	0.61 (0.36–1.01)
	Married	66 (19.8)	61 (18.3)	1	1
Occupation	Traders	86 (25.7)	55 (16.5)	0.41 (0.22–0.78)*	0.32(0.15-0.69)*
	Government employs	28 (8.4)	25 (7.5)	0.58 (0.27–1.23)	0.54 (0.23–1.3)
	Daily labors	46 (13.8)	38 (11.4)	0.54 (0.27–1.06)	0.5 (0.24–1.1)
	Students	22 (6.6)	34 (10.2)	1	1
Income (ETB)	Less than 1,000	50 (15)	31 (9.3)	0.72 (0.36–1.5)	0.37 (0.16–0.86)*
	1,000-2000	31 (9.3)	31 (9.3)	1.17 (0.6–2.44)	0.64 (0.28–1.5)
	2001–4,000	44 (13.2)	46 (13.8)	1.22 (0.62–2.4)	0.9 (0.4–2.0)
	4,001–6,000	29 (8.7)	20 (6)	0.81 (0.37–1.8)	0.58 (0.25–1.4)
	> 6,000	28 (8.4)	24 (7.2)	1	1
Khat chewing	Yes	94 (28.1)	73 (21.9)	0.86 (0.56–1.3)	0.91 (0.53–1.5)
	No	88 (26.3)	79 (23.7)	1	1
Frequency of khat chewing per day	≥ 3 times	43 (12.9)	27 (8.1)	0.68 (0.38–1.2)	0.73 (0.38–1.4)
	1–2 times	51 (15.3)	46 (13.8)	0.99 (0.6–1.6)	1.6 (0.8–3.0)
	None	88 (26.3)	79 (23.7)	1	1
Cigarette smoking	Yes	44 (13.2)	18 (5.4)	0.42 (0.23–0.77)*	0.4 (0.2–0.73)*
	No	138 (41.3)	134 (40.1)	1	1

*Statistical significant. ETB, Ethiopian birr.

#### Propensity score analysis

In this study, the logit model was used to predict propensity scores for the intervention group (khat chewing) in the study participants. The average propensity score was 0.5, with a standard deviation (SD) of 0.31 between the intervention group (khat chewing) and the control group (non-khat chewing). This value represents the average estimated probability of receiving treatment (khat chewing) based on observed factors. The baseline characteristics such as age, sex, religion, ethnicity, smoking, and shisha were significantly associated with khat chewing (Table 5).

**Table 5:** Logit regression analysis of factors associated with khat chewing.

Variables	Khat chewing	
	Coefficient	*p*-value
Age	0.059	0.008
Sex	0.74	0.016
Marital status	−0.03	0.92
Occupation	0.7	0.63
Religious	1.4	0.004
Ethnicity	1.07	< 0.001
Educational status	−0.24	0.13
Income	0.0001	0.097
Smoking	0.8	0.073
Alcohol	−0.0155	0.98
Shisha	2.8	< 0.001
Constant	−7.02	< 0.001

### The effect of khat chewing on sexual desire

We estimated the impact of khat chewing on sexual desire using the estimated difference sexual desire between the treated groups (khat chewers) and the matched control groups (non-khat chewers). The propensity score matching analysis estimates the impact of treatment (khat chewing) by controlling the background variables which have an association with sexual desire. A radius matching with a caliper width of 0.15 had the best matching quality and was used to estimate the ATE of khat chewing among participants, ATT and average effect among the untreated. The unmatched estimate showed that khat chewing among adults resulted in a 4.2% reduction in the level of sexual desire. The ATE of khat chewers was 18.9%, indicating that adults who chew khat have increased sexual desire by 18.9%. The ATT was 24.4%, indicating that adults with khat chewing had an increased sexual desire by 24.4% among treated groups (Table 6).

**Table 6:** Propensity score-matched analysis of the effect of khat chewing on sexual desire.

The effect of khat chewing on sexual desire	Treated (%)	Control (%)	Difference (%)	SE	*t*-statics
Unmatched	43.1	47.3	−4.2	0.055	−0.77
ATT	43.1	18.6	24.4	0.11	2.25
ATU	47.3	60.5	13.2		
ATE			18.9		

### Quality of matching

**Figure 1:**
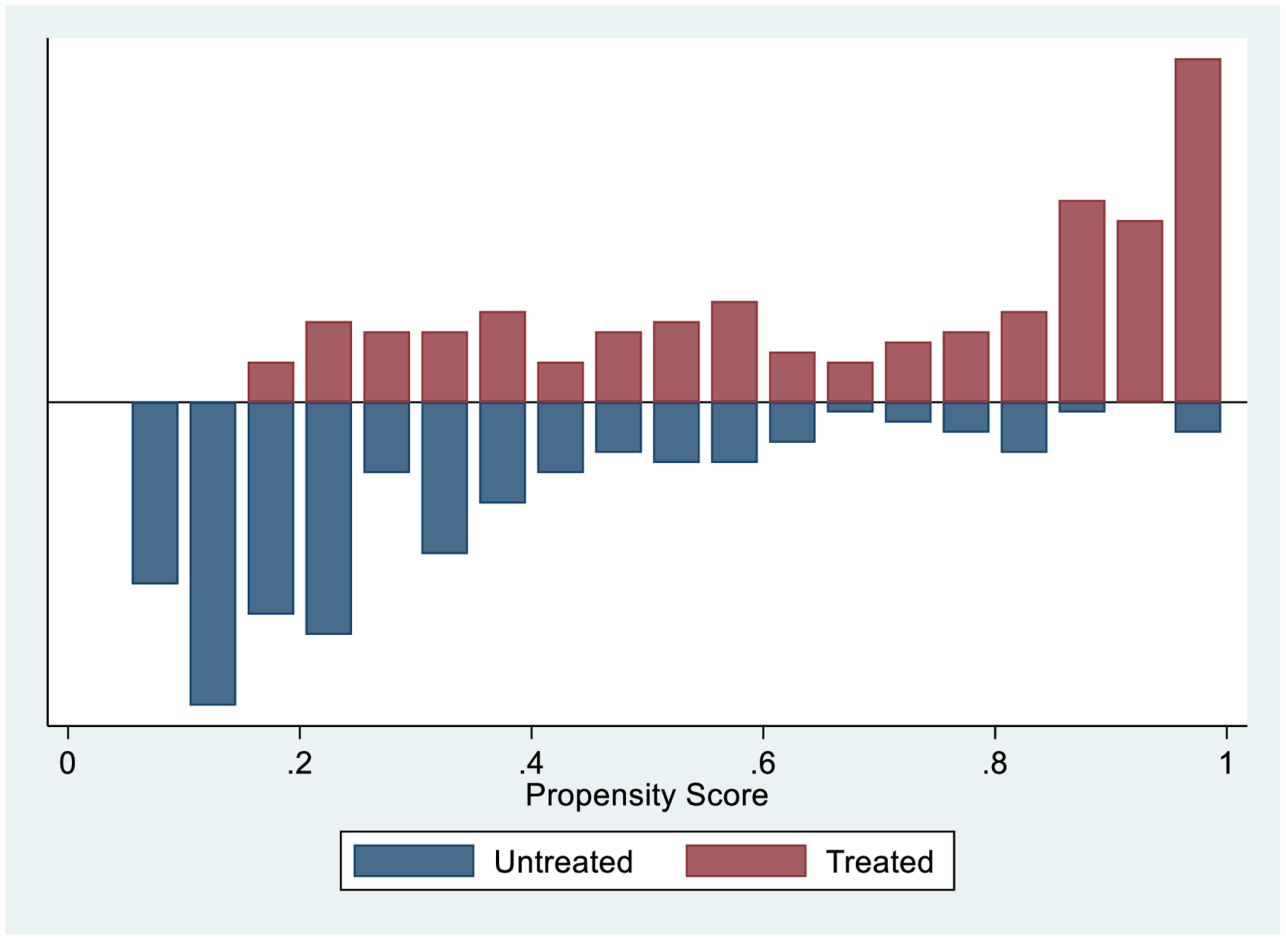
Histogram to show common support.

Common support assumptions were assessed using statistical tests (Tables 6–8) and graphically (Figure 1). Observations in the intervention and control groups with propensity scores outside the common support area were excluded from the analysis.

**Table 7:** Propensity score test to assess quality of matching.

Sample	Ps R2	LR chi2	p > chi2	Mean Bias	Med Bias	B	R
Unmatched	0.315	146.05	< 0.001	41.9	36.3	148.8*	2.16
Matched	0.099	45.8	< 0.001	22.8	19.5	73.2*	1.62

*indicates statistical significance.

**Table 8:** The common support.

Treatment assignment	On support	Total
Untreated	167	167
Treated	167	167
Total	334	334

#### Propensity score test

The pseudo-R2 indicates how well the covariates explain the participation probability. After matching, there should be no systematic differences in the distribution of covariates between the treatment (khat chewing) and control (non-khat chewing) groups. Thus, the pseudo-R2 should be relatively low. Standardized bias ranging between 3 and 5% post-matching is deemed sufficient (26). For the unmatched sample, the maximum absolute bias (B) is 148.8%, which is quite high, and the maximum absolute bias reduction (R) is 2.16. For the matched sample, the maximum absolute bias (B) is reduced to 73.2%, and R is 1.62, indicating a significant improvement in the balance of the covariates after matching (Table 7).

#### Common support

We included both untreated and treated groups when fitting the propensity-matched analysis of the effect of chewing khat on sexual desire because there was no off support from either group (Table 8). A histogram was plotted to visualize the distribution of propensity scores. The common support assumption was validated by the substantial overlap in characteristics between the treatment and control groups. The distribution is nearly identical for the post-matching propensity scores of both groups (Figure 1).

### Sensitivity analysis

We employed the Rosenbaum bounding approach to find out how much unmeasured variables (hidden bias) affected the matching analysis’s results and the selection process. Looking at the bounds under the assumption of overestimating the treatment effect, i.e., Q + MH, revealed small levels of *Γ*, which become insignificant at all values of Γ. More specifically, at both the 5 and 10% significance levels, the result was no longer significant for all values of Γ. According to this result, the odds ratio of treatment assignment between the treatment group (khat chewers) and comparison group (non-khat chewers) might differ by Γ values ranging from 1 to 2, but in that case, the confidence interval for the effect would include zero. The underestimate of the true treatment effect is insignificant under Γ = 1.0.1.25 and becomes increasingly significant as Γ increases (Table 9).

**Table 9:** Quality of matching for the effect of khat chewing on sexual desire.

Gamma (Ґ)	Test statistics		Significance level	
	Q_mh+	Q_mh-	p_mh+	p_mh-
1	1.09398	1.09398	0.136983	0.136983
1.05	0.973631	1.2241	0.16512	0.110457
1.1	0.854489	1.34382	0.196417	0.089504
1.15	0.740779	1.45842	0 0.229414	0 0.072362
1.2	0 0.632016	1.56836	0 0.263688	0 0.058399
1.25	0.527779	1.67402	0 0.298826	0 0.047064
1.3	0.427696	1.77573	0 0.334436	0 0.037888
1.35	0 0.331439	1.87381	0 0.370156	0 0.030478
1.4	0 0.238719	1.96852	0 0.405662	0 0.024504
1.45	0 0.149277	2.0601	0 0.440668	0.019694
1.5	0 0.062881	2.14877	0 0.474931	0 0.015826
1.55	−0.020677	2.23471	0 0.508248	0 0.012718
1.6	−0.101582	2.3181	0 0.540456	0 0.010222
1.65	−0.180006	2.39911	0 0.571426	0 0.008218
1.7	−0.137858	2.47786	0 0.554824	0 0.006609
1.75	−0.064452	2.55451	0 0.525695	0 0.005317
1.8	0 0.006881	2.62915	0 0.497255	0 0.00428
1.85	0 0.07626	2.70191	0 0.469606	0 0.003447
1.9	0.143793	2.77289	0 0.442832	0 0.002778
1.95	0 0.209579	2.84218	0 0.416998	0 0.00224
2	0.273711	2.90986	0.392153	0.001808

Gamma-: odds of differential assignment due to unobserved factors; Q_mh+−: Mantel–Haenszel statistic (assumption: overestimation of treatment effect); Q_mh-: Mantel–Haenszel statistic (assumption: underestimation of treatment effect); p_mh+−: significance level (assumption: overestimation of treatment effect); p_mh-: significance level (assumption: underestimation of treatment effect).

## Discussion

In this study, more than half of the respondents (54.5%) had poor sexual desire. Among khat chewers, 28.1% had a reduction in sexual desire. This proportion is less than in a study conducted in Yemen, which reported that 81% of erectile dysfunction cases were khat chewers (14). This difference could be the result of an imbalance of intrinsic and extrinsic factors among participants.

Younger adults and those with high-income levels had a higher sexual desire. The sexual desire of those aged 18–25 years old was higher compared to those aged 41–49 years old. Adults with low incomes had less sexual desire than adults with high incomes. These findings are in line with a previous study (27). The level of sexual desire among students was higher than that of traders, which may be due to their young age. Cigarette smokers had a low sexual desire compared to nonsmokers. These findings were contradicted with the previous studies’ findings (7–9). This difference may be related to a discrepancy in outcome measurements. In the previous study (7–9), the outcome was risky sexual behavior, while in the present study, the outcome was sexual desire. In this study, khat chewing did not significantly affect sexual desire. This finding is consistent with a previous study in Yemen that found no significant association was noted between khat chewing and psychological erectile dysfunction (14). However, according to earlier studies on human and animal models, khat increased sexual behavior or motivation (2, 7–13); conversely, other previous studies revealed that khat reduced libido (16–19). Variations in the study participants and the measurement of outcomes in the current and previous studies could account for this discrepancy. The cause of these contradictory findings between the studies may be the fact that the study subjects in the current study chewed khat during the last 30 days, while the previous study reported chronic khat chewers. The duration of exposure can have a significant impact on sexual desire. Another explanation would be that the dose of khat consumed may affect sexual behavior (15). The amount of khat consumed in the current study was determined by the frequency of chewing per day. However, the amount of khat consumed in a single chewing session was not measured.

Adult khat chewers had different baseline demographics, socioeconomics, related substance use, and sexual desire characteristics than adults who do not chew khat. Determining the relationship between khat chewing and sexual desire in comparison to not chewing using standard regression analysis would be biased regarding the impact of khat chewing since it would not account for observed selection bias. However, we used propensity score matching to account for selection bias in the observed variables in order to evaluate the true effect of khat chewing on sexual desire. In the propensity score matching analysis, the average treatment effect (ATE) of khat on sexual desire among chewers was 18.9%, meaning that adults with khat chewing had an increased sexual desire on average of 18.9%. The average treatment in the treated (ATT) group was 24.4%, which also indicated it increased sexual desire by 24.4% among treated groups (khat chewers). In other words, the current finding implies that khat chewing increases rather than decreases sexual desire. This evidence is consistent with the findings of previous studies, which reported that khat chewing increased sexual behavior (2, 7–13). However, comparing the findings of the current study with the previous studies (2, 7–13) poses a challenge because the definitions of risky sexual behavior and sexual desire are not the same. Furthermore, as some studies have been conducted in animal models, the comparison of animal sexual behavior with human sexual desire may not be accurate due to differences in sexual desires. On the other hand, the current finding is in contrast with previous studies that reported reduced libido in chronic or regular khat chewers (16–19). This variation may be caused by the definition of outcome in the current study, sexual desire, which is defined as an interest in engaging in sexual activity, and the type of activity that one feels a desire for (21). This concept includes sexual activity as well as interest in engaging in sexual desire and feeling sexual desire. However, in previous studies (16–19), impotency was a reduction in sexual activity, not a desire, interest, or feeling. An alternative rationale for the discrepancy could be the variations in research methodology, participant characteristics, and length of khat exposure.

### Strength and limitations of the study

Even though this study offers important insight into the actual effect of khat chewing on sexual desire, the result should be interpreted in light of the following limitations: The matching was done based on the observed variables only, and there may be residual confounding (unobserved variables). Sexual desire is a sensitive issue. Therefore, sociocultural pressures may exist on the expression. Due to the high temperature of the area, sexual desire may also be affected by this temperature. The duration of khat chewing that may affect sexual desire was not assessed. Recall bias could occur due to the nature of a cross-sectional study. Despite the abovementioned limitations, this study has the following advantages: the findings can be used for establishing harm reduction programs, and policymakers may be able to use the data to establish regulations and laws on the issue. The study could benefit khat users, researchers, clinicians, and the community as a whole, and it may serve as baseline data or a source of information for further studies, mainly for academicians and researchers to develop theories and sound screening tools for the effect of khat chewing on sexual desires. Moreover, this study adjusts potential confounders using the propensity score matching analysis in the estimation of the association between khat chewing and sexual desire. The estimations indicated a good quality of matching and were insensitive to hidden bias.

## Conclusion

More than half of adults had poor sexual desire. Logistic regression analysis revealed that sexual desire was moderated by being young, having a high income, and smoking cigarettes. Khat chewing did not significantly affect sexual desire. In the propensity score matching analysis, khat chewing increased sexual desire. Thus, khat chewer community should receive comprehensive sexuality education in order to address the effect of khat chewing on sexual desire and alter concerns about the relationship between khat and sexual desire. Additionally, the Ministry of Health implemented a harm reduction program in order to address the issues.

## Data Availability

The raw data supporting the conclusions of this article will be made available by the authors, without undue reservation.
