# Supplementary material for: The effect of khat chewing on sexual desire among adults in North East Ethiopia: a propensity score-match analysis
Source: Front Public Health. 2025 Jul 3;13:1470982. doi: 10.3389/fpubh.2025.1470982 (PMC12269856; doi:10.3389/fpubh.2025.1470982)
Supplement: Supplementary file 1 [file Table_1.DOCX]

Additional file 1: Sexual Desire Inventory

This questionnaire asks about your level of sexual desire. By desire, we mean *interest in or wish for sexual activity*. For reach item, please circle the number that best shows your thoughts and feelings. Your answers will be private and anonymous.

1. During this last month, *how often* would you *have liked* to engage in sexual activity with a partner (for example, touching each other’s genitals, giving or receiving oral stimulation, intercourse, etc.)?
   1. Not at all
   2. Once a month
   3. Once every two weeks
   4. Once a week
   5. Twice a week
   6. 3 to 4 times a week
   7. Once a day
   8. More than once a day
2. During this last month, *how often* have you had sexual thoughts involving a partner?
   1. Not at all
   2. Once a month
   3. Once every two weeks
   4. Once a week
   5. Twice a week
   6. 3 to 4 times a week
   7. Once a day
   8. More than once a day
3. When you have sexual thoughts *how strong* is your desire to engage in sexual behavior with a partner?

| 0 | 1 | 2 | 3 | 4 | 5 | 6 | 7 | 8 |
| --- | --- | --- | --- | --- | --- | --- | --- | --- |
| No desire |  |  |  |  |  |  |  | Strong desire |

1. When you first see an attractive person, *how strong* is your sexual desire?

0 1 2 3 4 5 6 7 8

No desire Strong desire

1. When you spend time with an attractive person (for example, at work or school), *how strong* is your sexual desire?

| 0 | 1 | 2 | 3 | 4 | 5 | 6 | 7 | 8 |
| --- | --- | --- | --- | --- | --- | --- | --- | --- |
| No desire |  |  |  |  |  |  |  | Strong desire |

1. When you are in romantic situations (such as a candle-lit dinner, a walk on the beach, etc.) *how strong* is your sexual desire?

| 0 | 1 | 2 | 3 | 4 | 5 | 6 | 7 | 8 |
| --- | --- | --- | --- | --- | --- | --- | --- | --- |
| No desire |  |  |  |  |  |  |  | Strong desire |

1. *How important* is it for you to fulfill your sexual desire though activity with a partner? 0 1 2 3 4 5 6 7 8

Not at all important Extremely important


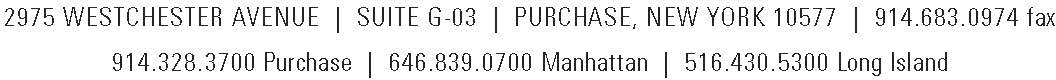


1. Compare to other people of your age and sex, how would you rate your desire to behave sexually with a partner?

| 0 | 1 | 2 | 3 | 4 | 5 | 6 | 7 | 8 |
| --- | --- | --- | --- | --- | --- | --- | --- | --- |
| Much less desire |  |  |  |  |  |  |  | Much more desire |

1. During this last month, *how often* would you have liked to behave sexually by yourself (for example, masturbating, touching you genitals, etc.)?
   1. Not at all
   2. Once a month
   3. Once every two weeks
   4. Once a week
   5. Twice a week
   6. 3 to 4 times a week
   7. Once a day
   8. More than once a day
2. *How strong* is your desire to engage in sexual behavior by yourself?

0 1 2 3 4 5 6 7 8

No desire Strong desire

1. *How important* is it for you to fulfill your desires to behave sexually by yourself? 0 1 2 3 4 5 6 7 8

Not at all important Extremely important

1. Compared to other people your age and sex, how would you rate your desire to behave sexually by yourself?

| 0 | 1 | 2 | 3 | 4 | 5 | 6 | 7 | 8 |
| --- | --- | --- | --- | --- | --- | --- | --- | --- |
| Much less desire |  |  |  |  |  |  |  | Much more desire |

1. *How long* could you go comfortably without having sexual activity of some kind?
   1. Forever
   2. A year or two
   3. Several months
   4. A month
   5. A few weeks
   6. A week
   7. A few days
   8. One day
   9. Less than one day


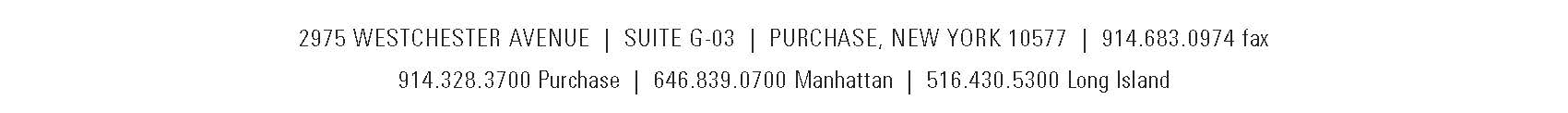


Source. This inventory was originally published in “The Sexual Desire Inventory: Development, Factor, Structure, and Evidence of Reliability,” by I. P. Spector, M. P. Carey, and L. Steinberg. 1996, Journal of Sex & Marital Therapy, 22, 175-190.
